# Detection of JC Polyomavirus tumor antigen in gastric carcinoma: a report from Iran

**Published:** 2018-08

**Authors:** Samira Izi, Masoud Youssefi, Farzad Rahmani, Nema Mohammadian Roshan, Atefeh Yari, Farnaz Zahedi Avval

**Affiliations:** 1 Department of Clinical Biochemistry, Faculty of Medicine, Mashhad University of Medical Sciences, Mashhad, Iran; 2 Department of Microbiology and Virology, Faculty of Medicine, Mashhad University of Medical Sciences, Mashhad, Iran; 3 Antimicrobial Resistance Research Center, Mashhad University of Medical Sciences, Mashhad, Iran; 4 Department of Pathology, Ghaem Hospital, Faculty of Medicine, Mashhad University of Medical Sciences, Mashhad, Iran; 5 Metabolic Syndrome Research Center, Mashhad University of Medical Sciences, Mashhad, Iran

**Keywords:** Oncogenic virus, Tumor antigen, Tumor suppressor proteins, Real-time PCR

## Abstract

**Background and Objectives:**

Factors contributing to development of gastric cancer are still under investigation. The JC Virus (JCV), as an oncogenic virus, has been indicated to play a possible role in gastric carcinogenesis. Theoretically, tumor antigen (T-Ag), the viral transforming protein, is capable of binding and inactivating tumor suppressor proteins p53 and pRb, there by promoting cancer development although such a role in gastric cancer is still controversial and additional data is needed to reach a definite conclusion. The prevalence of the virus varies in different geographic regions, therefore, we aimed to investigate JCV presence in cancerous gastric tissues of Iranian patients.

**Materials and Methods:**

Thirty-one paired samples were included in this study (total of 62 samples). T-Ag sequences were investigated using real-time PCR in formalin fixed paraffin embedded (FFPE) tissue samples from the tumor site and relevant adjacent non-cancerous tissues (ANCT). In positive samples, JCV copy number (viral load) was also measured using real-time PCR. To evaluate T-Ag protein expression, immunohistochemistry examination was performed using an anti-T-Ag specific antibody.

**Results:**

JCV sequences were detected in 17 out of 31 gastric cancer tissue samples (54.84%) and in 10 out of 31 of the non-cancerous adjacent gastric mucosa (32.25%) (Odds ratio of 2.4). Viral load in tumoral and adjacent tissue samples was not statistically different (p=0.88). Immunohistochemical study confirmed presence of JC T-Ag in the nuclear compartment.

**Conclusion:**

We showed the presence of the JC virus in gastric carcinoma tissue samples in our geographic region. This finding provides supportive data for a possible contribution of JCV in gastric cell transformation to malignancy. However, we highly recommend additional investigations to further explore JC virus and gastric cancer in order to reach a conclusion.

## INTRODUCTION

Although the global incidence and mortality rate of gastric cancer has decreased dramatically over the past decades, gastric cancer remains a major public health problem in many parts of the world (1, 2). Gastric carcinogenesis is a multistep and multifactorial process. The modification in function of genes that control epithelial cell growth and differentiation is an important key factor in the progression of gastric cancer (3). Exposure to biological and chemical carcinogens, such as *Helicobacter pylori*, oncoviruses, nitrosamines and oxidants which lead to DNA damage are the main known risk factors of gastric cancer (4).

In order to identify the predisposing factors contributing to development of cancers, some infectious agents such as *Epstein–Barr* virus (EBV) and *John Cunningham* virus (JCV) have been blamed to play a possible role in different stages of carcinogenesis process (5, 6). JCV belongs to the *Polyomaviridae* family and is a common human infection (7). The JCV genome is 5.13-kb, double-stranded, supercoiled circular DNA. JCV genome consists of early and late coding regions that are separated by a transcription control region (TCR) containing the promoter and enhancer elements for early and late transcription. Among these genes, two oncoproteins, large T antigen (T-Ag) and small t antigen (t-Ag), have been shown to be transforming and oncogenic proteins in experimental systems (8, 9).

Regardless of PML *(Progressive Multifocal Leukoencephalopathy)*, a fatal CNS complication in immunocompromised patients (10–12), infection with the JC virus has been considered subclinical in humans. However, it has been recently proposed that persistent infection with JC virus might be a potential risk factor for human carcinogenesis (6, 13–15). All *Polyomaviruses* have the ability to encode a version of large tumor antigen (T-Ag) that is a multifunctional protein capable of promoting transformation of cells through several pathways. T-Ag can modulate cellular signaling pathways and thereby induces the host cells to enter the S-phase, considering its function as ATPase, helicase, polymerase and DNA binding capacity; all of which are essential for DNA replication (16, 17).

Also, this protein has the ability to bind and inactivate tumor suppressor proteins p53 and the cell cycle regulator retinoblastoma gene product, pRb, leading to their impaired function (18–23). In addition to T-Ag transforming function, the JC viral genome may integrate into the host genome which in turn may cause genetic instability leading to malignant cells (18–20). Overall, different cellular mechanisms including rapid division, prolonged life span, enhanced production of plasminogen activator, anchorage-dependent growth, and unstable multicentric chromosome have been proposed for JC T-Ag carcinogenicity (24). Nevertheless, the role of JCV in gastric cancer is still controversial. Few reports provided supportive data on such a role though additional data are still needed to reach a definite conclusion. Here we investigated the presence of JCV T-Ag DNA sequences and its expression in gastric cancer and its non-cancerous adjacent tissue in Iranian patients.

## MATERIALS AND METHODS

### Samples.

Formalin fixed paraffin embedded (FFPE) tissue sample pairs of gastric cancer and adjacent non-cancerous tissue (ANCT) were obtained from the archives of the pathology department of Ghaem University Hospital, Mashhad, Iran. Samples from the past two years were included to obtain better preserved genome. The FFPE samples belonged to 18 men and 13 women diagnosed with gastric carcinoma.

### DNA extraction and real-time PCR.

FFPE tissue samples were deparaffinized and then genomic DNA was extracted. Firstly, one ml xylene was added to 25 mg of slice sample in a microcentrifuge tube and incubated for 30 min at 56°C until the paraffin was completely dissolved. The samples were centrifuged at full speed for 5 min and the supernatant was removed. In the next step, one ml ethanol (96–100%) was added to the deparaffinized tissue sample and gently mixed. After centrifuging and removing the supernatant solution, samples were incubated at 37°C for 15∼20 min to evaporate ethanol residue completely.

Then, genomic DNA was extracted from deparaffinized tissues in a clean pre-PCR room using the QIAamp DNA mini kit (Qiagen Inc., Valencia, CA) according to the manufacturer's instructions with slight modifications. After extraction, DNA was quantitated spectrophotometrically and kept at −20°C until used for real-time PCR assay.

Before real-time PCR, 400 ng of DNA was treated with 0.26 unit of topoisomerase I (BioLabs M0301S) for 45 minutes at 37°C to enhance primer access to supercoiled JC genomic DNA. For detection of JCV T-Ag sequence, SYBR Green Real Time quantitative PCR was applied using specific primers for T-Ag to produce a 171-bp amplicon related to the conserved N-terminal region of JCV T-Ag. PCR primers were designed using Beacon Designer software. Mad-1 reference sequence from National Center for Bio-technology Information (NCBI) reference genome (accession number NC: 001699.1) (www.ncbi.nlm.nih.gov) was used for primer design. The primer set used in this study was specific for the T-Ag of JCV, distinct from the T-Ag sequences of the homologous viruses such as SV40 and BKV. The primer pairs used in this study are shown in Table 1.

**Table 1.** Primer sequences for amplification of JC Virus T-Ag.

**Primer name**	**Primer sequence**	Primer position^*^
JCV T-Antigen F	5′-GGA TTA GTG GCA CAG TTA GG -3′	4700-4719
JCV T-Antigen R	5′-CGA AGA CAA GAT GAA GAG AAT G -3′	4870-4849

* Based on JC virus sequence Genbank

The qPCR amplification was performed in a Light Cycler instrument (Roche Applied Science). The PCR reaction mixture volume was 20 μL consisting of 1.5 μL of template DNA, 10 μL of Takara SYBR Premix Ex Taq II, 6.5 μL dH2O and 1 μL (10 pmol) of each primer. The following PCR protocol was used: initial denaturation step (95°C for 7 minutes), amplification and quantification program repeated 35 times (denaturation at 95°C for 15 seconds, followed by annealing at 60°C for 45 seconds and extension 72°C for 60 seconds with a single fluorescence measurement).

To prepare the positive control, the JCV (Mad-1) T-Ag gene was sub-cloned into pBHA plasmid vector and serially diluted to prepare the standard curve to be used for genome quantification calculation. Master Mix without template DNA served as a negative control. Beta-2 microglobulin (β2-MG) DNA was used as internal control of PCR reactions and reagents/instrumentation efficacy. Melting curve analysis was performed to confirm the specificity of reactions.

**Fig. 1.**
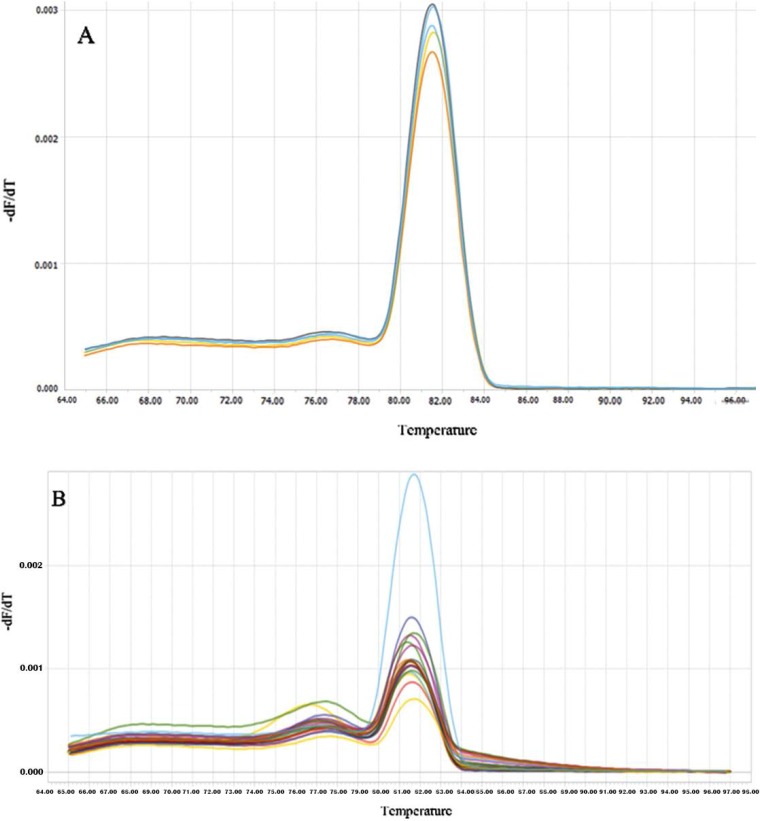
**Melting curve analysis.** (A) Amplicons from reference gene. The single peak observed is typically interpreted as representing a pure, single amplicon. (B) Amplicons from samples. The specificity of amplicons was verified by melting curve analysis after 35 cycles and agarose gel electrophoresis. Fluorescence melting peaks for the T-Ag gene, no separate heteroduplex products were apparent. Specific signals had melting temperatures of 81.4 ± 0.05°C.

**Fig. 2.**
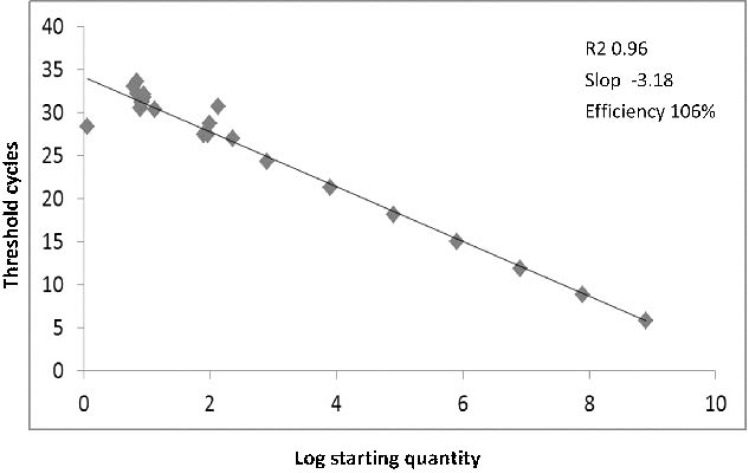
**Standard curve analysis.** Synthetic DNA standard curves for absolute quantification of JC-T-Ag by real-time PCR. The JCV-Mad-1 T-Ag sequence that cloned in a pBHA plasmid vector was serially diluted (from 100 to 10^8^ copies of positive control DNA) and served as a standard reference. High amplification efficiency has also been shown by a good linear relationship among each concentration.

Melt curve analysis was used in order to assess the specificity of qPCR products (Fig. 1). JCV-positive samples were identified by comparison of the test sample melt curves with the melt curves of the positive controls included in each run. The Ct value of the samples were measured and compared to the standard curve to calculate the viral T-Ag copy number (Fig. 2). The number copies was calculated according to the formula described by Rutledge and Stewart (25). Also, the PCR products were electrophoresed to evaluate the amplicon size and later subjected for sequencing. The clean-up process was done by the sequencing company.

### Immunohistochemical staining for T-Antigen.

Immunohistochemical staining was performed on the FFPE samples in 5 μm thick, mounted on positively charged slides. Briefly, tissue sections were placed in an oven at 65°C to melt the paraffin, followed by deparaffinization with xylene and rehydrated through serially gradient alcohol solutions. The sections subsequently were immersed into 0.01 M sodium citrate buffer (pH 6.0) and were heated intermittently in a microwave oven for 20 minutes to nonenzymatic antigen retrieval. Endogenous peroxidase activity was blocked with endogenous enzyme block solution (Dako Cytomation Inc., Carpinteria, CA) for 10 minutes.

The primary antibody used to detect specific T-Ag protein expression was a mouse monoclonal antibody against SV40 large T-Ag with cross reacting capacity with JCV T-Ag (clone pAb416, 1:100 dilution; Oncogene Research Products, San Diego, CA). Incubation of the primary antibody was performed overnight at 4°C and was followed by incubation in Dako EnVision+system-HRP polymer (Dako Cytomation Inc.) for 30 minutes. Staining was developed by reaction with diaminobenzide chromogen for 5 to 10 minutes. Next, slides were counterstained for 5 minutes with hematoxylin. The brown chromogen complexes were indicative of T-Ag-specific expression in the gastric tissues.

### Statistical analysis.

Demographic variables such as age and gender were entered in SPSS program version 21 for analyzing all data. Statistical evaluation was carried out using Wilcoxon Signed Ranks and MacNemar tests to differentiate non-parametric values. P-value less than 0.05 was considered statistically significant.

## RESULTS

### Viral T-Ag gene detection and JC quantification.

This study was performed on a set of biopsy specimens collected from gastric adenocarcinomas and, in parallel, from the healthy gastric mucosa surrounding the tumor mass from 31 patients including 18 men and 13 women diagnosed with gastric carcinoma and mean age of 64.90 ± 8.87 years.

JCV T-Ag sequences were found in 17 out of 31 (54.84%) neoplastic tissue samples and 10 out of 31 (32.25%) anatomically normal surrounding tissues (MacNemar Test, p=0.54). Only in 5 cases, both cancerous tissue and its adjacent non-cancerous tissue harbored T-Ag sequences. A paired Odds ratio of 2.4 (CI95%=0.84–6.81) was calculated.

Quantitative real-time PCR for T-Ag in paraffin embedded specimens was performed in order to compare viral copy number inside and outside of tumor. Standard curve was constructed using serial dilutions of JCV T-Ag positive control DNA plasmid (corresponding to 100 to 10^8^ copies) (Fig. 2). The number copies was calculated according to the formula described by Rutledge and Stewart (25). The viral DNA loads ranged from 2, 030 to 1.97 × 10^5^ copies/μg DNA (mean ± SD, Median, Interquartile range, 11252±35748, 2250, 0–3770) in tumor tissue, and 389 to 9.42 × 10^4^ copies/μg DNA (mean ± SD; Median, Interquartile range, 7934±18744, 0, 0–6150) in surrounding healthy tissue (Wilcoxon Test, p=0.88).

In order to check the specificity of the reaction, melting curve analysis was performed and a single peak without secondary configurations or nonspecific signals was observed. A peak related to β-2 microglobulin control was observed in all experiments indicating adequate integrity of the extracted DNA and efficacy of reaction.

**Fig. 3.**
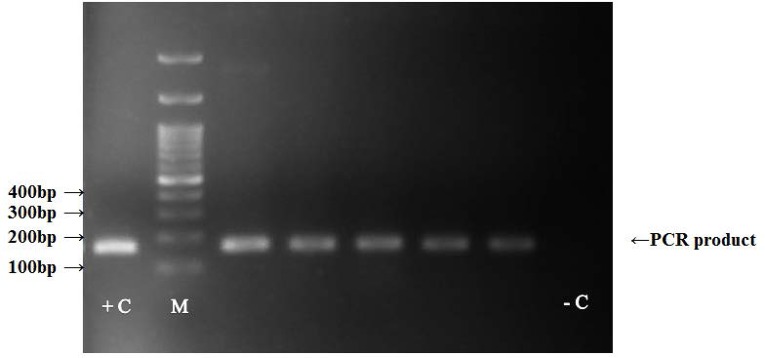
PCR amplification of T-antigen of JCV in gastric cancer and non-neoplastic tissues. A 171 bp bond corresponding to amplified sequences are shown. (+C; positive control, −C; negative control)

Next, the positive samples of PCR were subjected to gel electrophoresis and a 171 bp bond corresponding to the relevant amplicon size was observed (Fig. 3). Subsequently, T-Ag-positive PCR products were further confirmed by DNA sequencing and in each case, the presence of JCV and its specificity was validated using BLAST (Basic Local Alignment Search Tool) available from NCBI.

### Expression of oncogenic T-Ag protein.

**Fig. 4.**
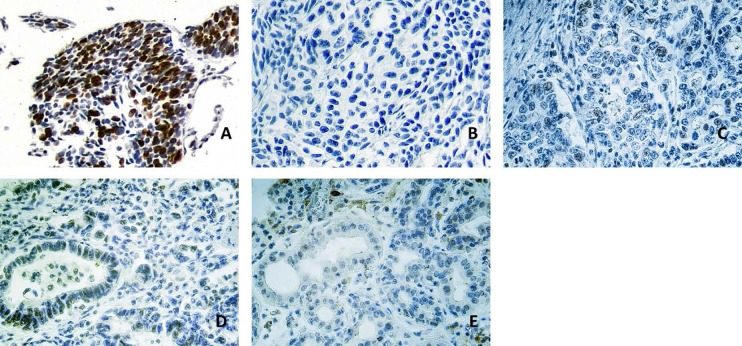
Panel A: Colon cancer cells infected by JC virus were used as a positive control, of which 20–30% cells were positive. (grade III); Panel B, C, D: These photomicrographs show immunohistochemical staining for JCV T-Ag in gastric tumor tissue samples. B. Tumor sample without expression of T-Ag. C. a weak and focal positive nuclear staining can be observed in the nuclei of tumor cells (shown in brown; grade I) D. positive staining in the nuclei of tumor cells (grade II); Panel E: Positive staining of grade I in the nuclei of endothelial cells of healthy mucosa surrounding a gastric adenocarcinoma. Original magnification ×400.

We performed immunohistochemical staining to show T-Ag expression in gastric cancer tissue. Fig. 4 illustrates one representative panel of positive and negative results. In positive samples, the reaction was visualized in the nuclear compartment of the cells (Fig. 4).

## DISCUSSION

In this study, JCV sequences were found in 54.83% of neoplastic tissue samples and 32.25% of anatomically normal surrounding tissue. These results are in agreement with our previous findings evaluating JCV in colorectal cancer (unpublished data); in addition, these observations are in line with data from other laboratories reporting the presence of JCV DNA sequences in the mucosa of the gastrointestinal tract, like colon cancer tissue, suggesting the possible role of polyomaviruses in development of tumor, mainly due to the presence of potent transforming genes such as large tumor antigen (T-Ag) (6, 14, 15, 26, 27). Analogous to other malignancies, gastric carcinogenesis is a multistage process with a multi-factorial etiology (28).

During the last few decades, there have been several reports emphasizing the association of Polyomavirus with different types of human carcinomas such as brain and colorectal cancers (13, 15, 27, 29, 30). JC virus strains worldwide can be classified into various genotypes based on DNA sequence variations. Although based on the structure of its TCR, JCV can be classified into two forms: the archetype and tandem repeat variants. Among the tandem repeat variants of JCV, the Mad-1 is the exclusive strain selected for replication and persistently infects the gastrointestinal tract. Several studies also have shown different single nucleotide polymorphisms (SNPs) in the T-Ag region of the JC virus (31, 32) though a conserved area of T-Ag sequence was used for primer design in this study.

To the best of our knowledge, this is the first report of JCV DNA in gastric tissues from Iranian patients, a region where gastric cancer is common. It should be noted that except for few reports, little is known about epidemiology of JC infection among the Iranian population (33). In the present study, the sensitivity of the method used was critical; immunohistochemical reactions were less sensitive probably due to T-Ag degradation in formalin-fixed and paraffin-embedded tissue samples. In positive samples, the protein was visualized in nuclei where it mediates its oncogenic role after its expression in the cytoplasm.

T-Ag is a multifunctional protein with the ability to bind and inactivate tumor suppressor proteins including pRb and p53. Different gene-based association studies explored the relationship between single nucleotide polymorphisms (SNPs) of oncogenes and tumor suppressor genes and various cancers such as gastric cancer and colorectal cancer in the Iranian population which should be noted in the interpretation of T-Ag oncogenic function (34, 35). It has been suggested that with these functions, T-Ag can inactivate cell cycle check points and induce the host cells to enter S-phase resulting in unchecking cellular replication (19–21, 23).

Experimentally, it is difficult to detect viral sequences in human tumors and more importantly, it is difficult to reproduce results in different laboratories. In some cases, detection rates for T-Ag in infected tumor cells might be reduced due to a low copy number. Some viruses can induce cellular integration and differentiation process involving genomic rearrangement and loss of the viral genome during the process called “hit-and-run”, which might have pathogenic roles in cancer progression (15). In this case, the target sequences might not be detectable by PCR. Also, a severely undifferentiated cancer tissue may be non-permissive for JCV replication. Technical reasons such as degraded extracted DNA as well as DNA quality could be other explanations for varying rates of JCV detection.

In the present study, the efficacy of our PCR reaction was enhanced by topoisomerase I pretreatment which has been recommended by previous studies in order to unwind supercoiled viral DNA genes resulting in improved access of the primers to their target sequences (36, 37). Investigators previously have used internal controls as a method to monitor the PCR amplification performance including extraction, amplification and detection steps. In this study, β-2 microglobulin (β2-MG) DNA served as a sensitive internal control (38, 39).

Due to high homology between JCVT-Ag with other polyomaviruses, including SV40 and BKV at the nucleotide level, our PCR primers were designed specifically to amplify only JCV T-Ag Mad-1Ref sequences (31). To ensure that our real-time PCR results were accurate; the specificity of T-Ag detection was confirmed by PCR electrophoresis, immunohistochemistry and sequencing. The results showed the presence of the virus in neoplastic tissues and non-cancerous tumor adjacent to the cancerous tissues though the viral load was not significantly different which is consistent with other reports (26, 39). Similar to previous studies in the gastrointestinal tract and in colon cancer, in the present study, we detected Mad-1 or type I-R form of JCV strain in gastric cancer tissue (31).

JCV might not necessarily be the main cause of gastric cancer but it can contribute, to some extent, to development of cancerous cells at one or several stages of tumor progression. To ensure the validity of the results, further molecular, cellular and *in vivo* investigations are encouraged to evaluate the role of T-Ag as a potential factor involved in pathways leading to the development or progression of cancer. Such studies with large numbers of cases at different stages, including different JCV strains and genotypes will be needed to address the efficacy and possible mechanisms in the field of JCV carcinogenesis.

Some limitations of this study should be noted. First, like many patient-based studies, lack of underlying confounding data should be mentioned. Second, like some other studies, DNA extraction efficacy was determined spectrophotometrically and we did not use a more accurate method of monitoring DNA extraction using extraction of defined number of plasmids. Also, extended sample size could be beneficial. Technically we were unable to include healthy gastric tissue samples. Although the presence of virus in a healthy control sample would not entirely rule out its possible role in tumorigenesis and *vice versa*, its absence in healthy controls cannot fully confirm the tumorigenicity, however, it could provide supporting data. It should be noted that in the present study, we showed the existence of the virus and we cannot conclude a cause and effect relationship. However, due to tumorigenic features of the virus, the observed association might support a causal relationship. Additional supportive data and multicentral longitudinal cause and effect studies are needed to make a definite conclusion.

To summarize, the current results clearly indicate the presence of JC polyomavirus sequence in human gastric mucosa independent to the copy number. The study is important from the point of view that it was the first attempt at detecting JCV in gastric carcinoma in the Iranian population and provides supportive data for possible role of JC in gastric cancer. Further investigations to explore the extent of its contribution to tumorogenesis is still needed.
